# No Seasonal Accumulation of Resistant *P. falciparum* when High-Dose Chloroquine Is Used

**DOI:** 10.1371/journal.pone.0006866

**Published:** 2009-08-31

**Authors:** Johan Ursing, Poul-Erik Kofoed, Amabelia Rodrigues, Lars Rombo

**Affiliations:** 1 Malaria Research, Department of Infectious Diseases, Karolinska Hospital, Karolinska Institutet, Stockholm, Sweden; 2 Projecto de Saúde de Bandim, Indepth Network, Bissau, Guinea-Bissau; 3 Department of Paediatrics, Kolding Hospital, Kolding, Denmark; 4 Research Initiative of Health Services, Kolding Hospital, Kolding, Denmark; 5 Department of Infectious Diseases, Mälarsjukhuset, Eskilstuna, Sweden; 6 Center for Clinical Research, Sormland County Council, Eskilstuna, Sweden; Walter and Eliza Hall Institute of Medical Research, Australia

## Abstract

**Background:**

Potentially chloroquine resistant *P. falciparum*, identified by the 76T haplotype in the chloroquine resistance transporter (*pfcrt* 76T), are highly prevalent throughout Africa. In Guinea-Bissau, normal and double dose chloroquine have respective efficacies of 34% and 78% against *P.falciparum* with *pfcrt* 76T and approximately three times the normal dose of chloroquine is routinely taken. Proportions of *pfcrt* 76T generally increase during high transmission seasons, as *P.falciparum* with *pfcrt* 76T commonly survive treatment with normal dose chloroquine. In Guinea-Bissau, there should be no seasonal increase of *pfcrt* 76T if the high doses of CQ commonly used are effective.

**Methods and Findings:**

*P. falciparum* parasite density, age, sex, the proportion of chloroquine resistance associated haplotypes *pfcrt* 76T and *P. falciparum multidrug resistance gene 1* 86Y were assessed in 988 samples collected from children between 2002 and 2007. There was no seasonal accumulation of any allele. During the high and low transmission periods the *pfcrt* 76T proportions were 24% (95% CI, 21–27%) and 26% (95% CI, 20–33%). There was no significant change of *pfcrt* 76T (OR 1.05, 95% CI; 0.94–1.16 p = 0.39) or *pfmdr1* 86Y (OR 0.92, 95%CI; 0.83–1.01 p = 0.08) proportions between 2003 and 2007. Lower median parasite density (*P.falciparum*/µl) was associated with *pfcrt* 76T (15254 [95% CI, 12737–17772]; n = 164) compared to *pfcrt* 76K (18664 [95% CI, 16676–20653]; p = 0.003; n = 591). Similarly, *pfmdr1* 86Y was associated with a lower median parasite density (16320 [95% CI, 13696–18944]; n = 224) compared to *pfmdr1* 86N, (18880 [95% CI, 16701–21059]; P = 0.018; n = 445).

**Conclusions:**

In contrast to the rest of Africa, *P. falciparum* parasites resistant to normal dose chloroquine do not have a selective advantage great enough to become the dominant *P.falciparum* type in Guinea-Bissau. This is most likely due to the efficacy of high-dose chloroquine as used in Guinea-Bissau, combined with a loss of fitness associated with *pfcrt* 76T.

## Introduction

Chloroquine resistant (CQR) *Plasmodium falciparum* spread through Africa during the 80's and 90's and was first described in Guinea-Bissau in 1990 [1]. Until June 2008 chloroquine (CQ) remained, by far, the most commonly used antimalarial in the country. In Guinea-Bissau, as in most other areas of Africa, CQR is associated with a mutation in the CQR transporter (*pfcrt* K76T) [2], [3]. Despite the presence of the *pfcrt* 76T mutation and continued CQ use that should select CQR *P. falciparum*, the prevalence of CQR *P. falciparum* is exceptionally low and unchanged in Guinea-Bissau [4].

The median CQ doses prescribed and reportedly taken in Guinea-Bissau were 81 and 77 mgkg^−1^, divided into 2–3 doses per day for median 5 days [5]. According to local physicians, CQ has always been prescribed in this way and use of high doses has been recorded since 1994 [4]. We have shown that 50 mgkg^−1^ of CQ in 2 divided daily doses over 3 days resulted in a 92% PCR corrected efficacy at day 28 [6] whereas treatment with the standard dose of 25 mgkg^−1^ given during 3 days had an 80% efficacy [6]. In addition, when treating *P. falciparum* carrying the CQR associated genetic marker (*pfcrt* 76T), 50 mgkg^−1^ had a 78% efficacy whilst only 34% were successfully treated with 25 mgkg^−1^ [2].

The marked reduction of the *pfcrt* 76T haplotype in Malawi following the withdrawal of CQ [7] suggests that *pfcrt* 76T identifies a less fit parasite than *pfcrt* 76K in the absence of CQ. In line with that, CQ resistance has been shown to be an energy dependent process [8]. Studies from Sudan and The Gambia reported increasing *pfcrt* 76T haplotype prevalence during the high transmission season and decreasing during the low transmission season [9], [10]. The probable explanation is that *pfcrt* 76T carrying parasites survive treatment with standard CQ doses, thereby accumulating during the high transmission season when CQ is frequently used. When less CQ is used during the low transmission season, haplotype prevalences are reversed because of better fitness of the 76K haplotype in the absence of CQ.

If the high dose CQ treatment commonly used in Guinea-Bissau is efficacious as our data suggests, *pfcrt* 76T carrying parasites will lose much of their survival advantage and therefore not accumulate markedly during the high transmission season. To evaluate this hypothesis we have analysed seasonal variations of the proportions of *pfcrt* 76K and 76T and *pfmdr1* 86N and 86Y in Guinea-Bissau.

## Methods

The studies were conducted at the Bandim Health Project in Bissau, Guinea-Bissau. The Bandim Health Project is a Demographic Survey Site (DSS) covering approximately 16 km^2^, mainly comprising semi-urban areas. The population is approximately 90 000 including about 12 000 children <5 years of age. Three primary health care centres, Bandim, Belem and Cuntum serve the population. In addition, the national hospital with 125 beds 5 km away, also serves as a primary contact as well as a referral hospital for patients.

Children develop symptomatic *P. falciparum* malaria infections all the year round in Guinea Bissau. However, there is a distinct seasonality with higher incidence of malaria between May and December just before, during and after the rainy season that lasts from May/June to October/November. The malaria prevalence has decreased over the years. In 1990, 183/312 (59%) of children (aged 3–6 yrs) had *P. falciparum* in community surveys during the rainy season compared to, 7/197 (3.6%) of children (aged<5 yrs) in 2004 [11].

Since October 2002 (except for Oct 2006) three clinical trials including collection of blood spotted onto filter-papers at day 0 for genotyping have been conducted back to back at Bandim Health Centre. From November 2006 until December 2008 the last study also recruited patients from Belem and Cuntum health centres. Inclusion criteria were similar in all studies requiring microscopically verified malaria (>20 *P. falciparum* per 200 white blood cells) with fever (or history of fever in the past 24 hours) in the absence of signs of severe malaria (convulsions, severe anaemia, hyper-parasitaemia, clinically poor condition).

Following informed consent a total of 988 (501 boys and 486 girls) children were included between October 2002 and December 2007. The total number of inclusions each month between January and December were 78, 49, 32, 32, 71, 118, 93, 66, 58, 137, 147, 107. We designated January – April as the low transmission season and May to December as the high transmission season. The high transmission season was split into early (May-August) and late (September-December) high transmission season. This split is halfway through the season and also at the point of lowest number of new cases during the high transmission season.

*Pfcrt* K76T, *pfmdr1* N86Y, *pfmsp1* and *pfmsp2* were identified using previously described PCR based methods [12], [13]. PCR and restriction products were resolved on 2% agarose gels (Amresco, Solon, OH). All gels were stained with ethidium bromide and visualized under UV transillumination (BioRad GelDoc System, BioRad, Hercules, CA).

Monthly rainfall data from 2003 to 2007 was provided by the National Meteorological Service and are presented as mean monthly values for the whole period.

Ethical approval was given by the Ministério da Saúde Pública in Guinea-Bissau (019/DHE/2004 and 030/DHE/2004 and 064/DGSP/2006), Karolinska Institute in Stockholm, Sweden (2005/111-31/1, 2009/881-31/4 and 2006/1151-31/1), and the Central Ethical Committee in Denmark (624-01-0042 and 2004-7041-11). The studies were registered at ClinicalTrials.gov (https://register.clinicaltrials.gov/) with the study IDs PSB-2001-chl-amo, NCT00137514 and PSB-2004-paracetamol, NCT00137566 and PSB-2006-coartem NCT00426439.

Age and parasite density over the years 2002–2007 were compared using the non-parametric test for trend. Genotyping data from October 2002 to December 2007 were pooled and monthly proportions of *pfcrt* 76K and 76T and *pfmdr1* 86N and 86Y were calculated. Changes in allele proportions by month or year were assessed using logistic regression. Because children were not recruited into the study throughout the year 2002, only data from 2003–2007 were used to assess any trend of changing annual haplotype proportion. Variations of parasite density were assessed by quantile regression using bootstrapping with 1000 repetitions. Haplotypes were analysed as 3 groups (76T, 76K or both for *pfcrt* and 86Y, 86N or both for *pfmdr1*). The association between *pfmsp* families and *pfcrt* and *pfmdr1* haplotypes were assessed using Fishers exact test.

## Results

### Study population

The median age was 65 months, the inter-quartile range (IQR) was 38-105 months and the median parasitaemia was 15600 (IQR 6800-38400) *P. falciparum* per micro-litre assuming a white blood cell count of 8000 per micro-litre (Table 1).

**Table 1:** Age, sex and parasitaemia by year.

Year	2002	2003	2004	2005	2006	2007	P(trend)
Nr. of children	103	333	158	171	115	108	
Sex male:female	57∶46	155∶178	83∶74*	85∶86	62∶53	58∶49	
Age in months	68 (34–117)	71 (40–108)$	60 (36–105)	64 (38–94)	64 (43–96)	60 (38–90)	0.07
*P. falciparum* per µl blood	14000 (7600–17200)	14400 (6400–18600)	16100 (4000–40000)	25000 (5520–40000)	34188 (11560–66667)	20000 (7970–66667)	<0.001

$ age missing for 4 children,

* sex unknown for one child.

Age and parasitaemia are presented as median values with interquartile ranges in brackets.

Data from 2002 is not included in presented p-values but inclusion of 2002 did not significantly alter the results.

### Stable proportions of pfcrt 76K and 76T and pfmdr1 86N and 86Y during the year

*Pfcrt* 76K and 76T alleles were successfully identified in 954/988 samples and *pfmdr1* 86N and 86Y alleles in 958/988. If both alleles were identified at one locus both were included as numerators but only counted as one in the denominator.

**Figure 1:**
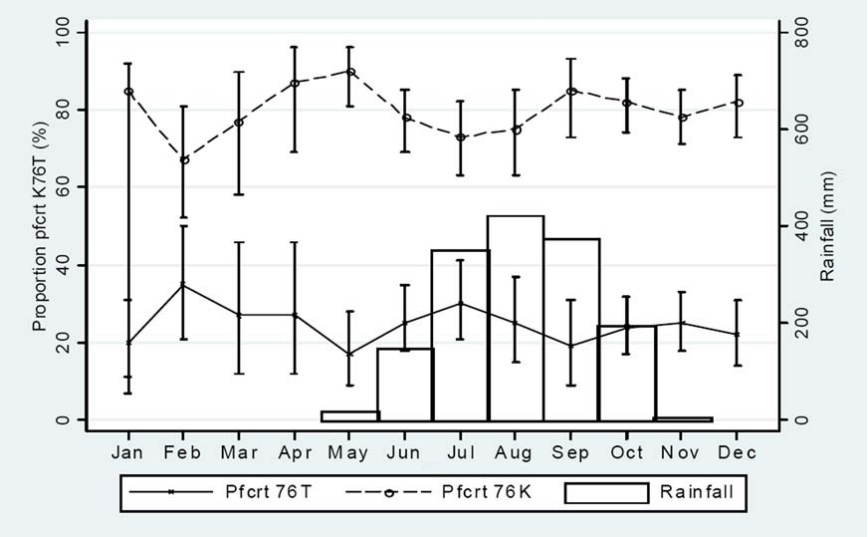
Monthly variation of *pfcrt* K76T haplotypes amongst children presenting with uncomplicated P. *falciparum* malaria between October 2002 and December 2007. Mean haplotype prevalence were calculated from samples collected continuously between October 2002 and December 2007. Binomial exact 95% confidence intervals are presented as range spikes with caps.

**Figure 2:**
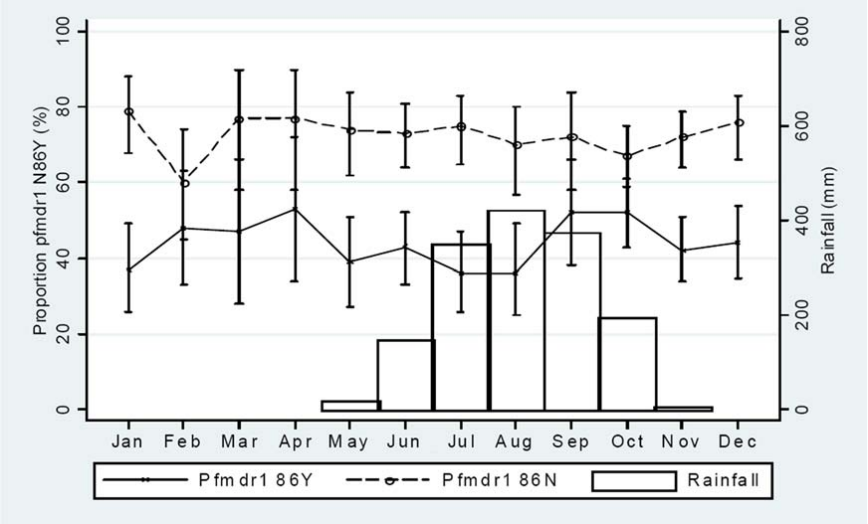
Monthly variation of *pfmdr1* 86N and 86Y haplotypes amongst children presenting with uncomplicated P. *falciparum* malaria between October 2002 and December 2007. Binomial exact 95% confidence intervals are presented as range spikes with caps.

There was no continuous monthly trend of changing *pfcrt* 76T or 76K (figure 1) nor *pfmdr1* 86Y or 86N (figure 2) allele proportions during the high (May-December) or the low (January-April) transmission periods. The total *pfcrt* 76T proportions during the high and low transmission periods were 186/777 (24% [95% CI, 0.21–0.27]) and 46/177 (26% [95% CI, 0.20–0.33]), respectively. The total *pfcrt* 76K proportions were 621/777 (80% [95% CI, 0.77–0.83]) and 140/177 (79% [95% CI, 0.73–0.85]), respectively. The total *pfmdr1* 86Y proportions were 338/779 (43%) and 79/179 (44%), respectively. The total *pfmdr1* 86N proportions were 562/779 (72%) and 131/179 (73%), respectively. There were no significant differences.

Though no marked seasonal differences were seen, there were minor fluctuations. From February to May there was a monthly decrease in the *pfcrt* 76T proportion (OR, 0.74 [95% CI, 0.56–0.98]; p = 0.04), whilst the *pfcrt* 76K proportion increased (OR, 1.65 [1.20–2.29]; p = 0.002) per month. From May to July there was a monthly increase of the *pfcrt* 76T proportion (OR, 1.43 [95% CI, 0.99–2.06]; p = 0.06), whilst the *pfcrt* 76K proportion decreased (OR, 0.59 [95% CI, 0.39–0.88]; p = 0.01) per month. There were similar but opposite trends from July to September but the changes were not significant.

### Proportions of alleles pfcrt 76K and 76T and pfmdr1 86N and 86Y between 2003 and 2007

**Figure 3:**
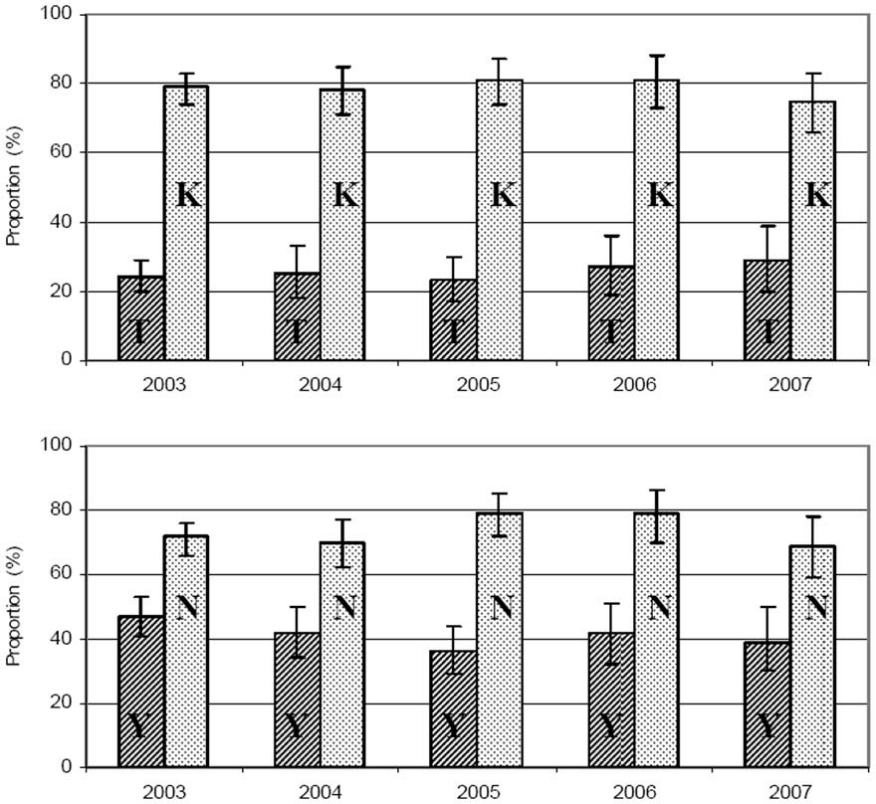
Proportion of *Pfcrt* 76K and 76T and *pfmdr1* 86N and 86Y haplotypes amongst children presenting with uncomplicated P. *falciparum* malaria from January 2002 to December 2007. T = *pfcrt* 76T, K = *pfcrt* 76K, Y = *pfmdr1* 86Y, N = *pfmdr1* 86N.

>For each of the years 2003–2007 *pfcrt* K76T and *pfmdr1* N86Y (in brackets if different) proportions are based on successful amplifications from 320 (319), 153 (157), 167 (168), 113 and 104 patients. There was no significant change of *pfcrt* 76T (OR 1.05, 95%CI; 0.94–1.16 p = 0.39), *pfcrt* 76K (OR 0.98; 95%CI, 0.88–1.11, p = 0.79), *pfmdr1* 86Y (OR 0.92, 95%CI; 0.83–1.01 p = 0.08) or *pfmdr1* 86N (OR 1.05, 95%CI; 0.94–1.16 p = 0.43) proportions between 2003–2007 (figure 3). The minimum and maximum haplotype proportions were as follows: *Pfcrt* 76T, 23–29%. *Pfcrt* 76K,75–81%. *Pfmdr1* 86Y, 36–47%. *Pfmdr1* 86N, 69–79%.

### Fluctuations in parasite densities during and between seasons

During early high transmission season parasite densities were higher compared to low transmission season irrespective of *pfcrt* K76T haplotype. For *P. falciparum* with 76K only, parasite densities during early high transmission season were also higher than during late high transmission season while there was no difference between late high and low transmission season. For *P. falciparum* with 76T only, parasite densities were higher during both early and late high transmission season compared to the low transmission season. (Table 2).

**Table 2:** Median number of *P. falciparum* per µl whole blood by season.

Samples analysed	*P. falciparum*/µl			
	Median	95% CI	P	Number
**All**				
January-April	13200	10965–15435		191^a^
May - August	*20833*	*19175–22491*	**<0.0001**	348^b^
September - December	*14800*	*14183–15417*	**0.16** *(<0.0001)*	449^c^
***Pfcrt*** ** 76T**				
January-April	7600	1196–14004		37
May - August	*20000*	*12093–27907*	**0.04**	73
September - December	*14400*	*11697–17103*	**0.05** *(0.31)*	83
***Pfcrt*** ** 76K**				
January-April	14400	12392–16408		131
May - August	*21053*	*16848–25258*	**0.003**	256
September - December	*15200*	*14087–16314*	**0.48** *(0.003)*	335

Includes 14^a^, 7^b^ and 13^c^ children for whom *pfcrt* K76T genotyping failed.

P values and confidence intervals were calculated by quantile regression (1000 repetitions).

P values in bold refer to differences compared to the low season January - April.

P values in brackets and italics refer to comparisons between seasons May-August and September –December.

Data from mixed infections are not presented as the numbers are small (9, 12 and 18 per season).

### Lower parasite densities were associated with pfcrt 76T and pfmdr1 86Y

For children under the age of 10 years, the age adjusted median parasite density (*P.falciparum*/µl) was lower when only *P. falciparum* with *pfcrt* 76T (15254 [95% CI, 12737–17772]; n = 164) compared to only *pfcrt* 76K (18664 [95% CI, 16676–20653]; p = 0.003; n = 591) was identified. Similarly, *pfmdr1* 86Y was associated with lower age adjusted median parasite density (16320 [95% CI, 13696–18944]; n = 224) compared to *pfmdr1* 86N, (18880 [95% CI, 16701–21059]; P = 0.018; n = 445). If the children over the age of 10 years were included in the analyses the differences were not significant but the trend the same. The median parasite density was 1977 parasites per µl less in children with *pfcrt* 76T compared to children with *pfcrt* 76K (p = 0.13) and 1504 parasites per µl less in children with *pfmdr1* 86Y compared to children with *pfmdr1* 86N (p = 0.11).

### Association between pfcrt K76T haplotype proportions, transmission rate and drug pressure (figure 4)

Through out the year the number of treatments and the proportion of *pfcrt* 76T vary in a remarkably similar fashion. The malaria incidence starts to increase between April and May just before the rainy season whilst the *pfcrt* 76T haplotype proportion start to increase approximately one month later. The malaria incidence peaks in June whilst the *pfcrt* 76T proportion peaks one month later.

**Figure 4:**
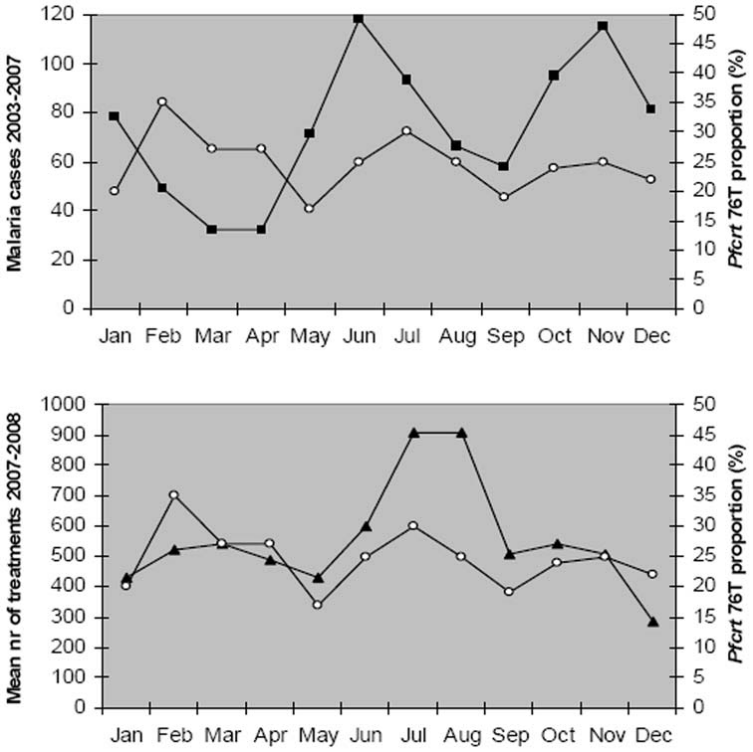
Monthly variation of *pfcrt* 76T proportion, monthly number of children with microscopy verified malaria and mean monthly number of children treated for malaria. Hollow circles. *Pfcrt* 76T prevalences in samples collected between October 2002 and December 2007. Black squares. The number of malaria cases (incidence) verified by microscopy in studies 2003 to 2007. Black triangles. The mean number of malaria treatments each month over the 2 year period December 2006 to November 2008.

### The number of genotypes per infection by season

**Figure 5:**
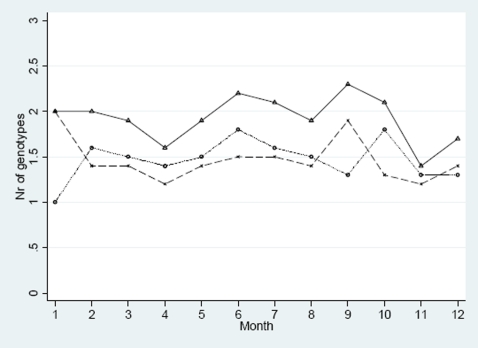
Monthly variation of the mean number of *pfmsp1* and *pfmsp2* genotypes. X = Pfmsp1, O = pfmsp2, Δ = nr of genotypes in both *pfmsp1* and *pfmsp2.*

A total of 163 samples from one complete year July 2004 – June 2005 were analysed. *Pfmsp1* and *pfmsp2* genotypes were successfully identified in 156/163 and 153/163 children, respectively (figure 5). The mean number of clones during the high and low transmission seasons were 1.96 [95% CI, 1.73–2.18] and 1.84 [95% CI, 1.47–2.21], respectively. There were no significant seasonal changes. The *pfmsp1* genotype R033 was identified in 42/46 samples that also carried *pfmdr1* 86N whilst only 4/46 R033 genotypes also had the *pfmdr1* 86Y haplotype (p = 0.01). No association was found between *pfmdr1* 86Y or *pfcrt* 76K or 76T with any of K1, MAD, Ro33, FC or IC genotypes.

### Linkage disequilibrium

Parasites carrying *pfcrt* 76T were more likely to also carry *pfmdr1* 86Y (OR, 2.71 [95% CI, 1.99–3.67]; P<0.0001; n = 951). Parasites carrying *pfcrt* 76K were more likely to also carry *pfmdr1* 86N (OR, 3.40 [95% CI, 2.45–4.74]; P<0.0001; n = 951). There was no seasonal variation.

## Discussion

We present continuously collected data from clinical trials with the same basic inclusion criteria conducted between October 2002 and December 2007. The proportions of *pfcrt* 76K and 76T and *pfmdr1* 86N and 86Y do not vary significantly between high and low transmission season in Guinea-Bissau and the proportions remained unchanged between 2003 and 2007.

The low and stable proportion of *pfcrt* 76T indicates that this haplotype (and thus *P. falciparum* normally resistant to standard dose CQ) does not have a selective advantage great enough to become the dominant haplotype in Guinea-Bissau. We have previously shown that double normal dose CQ has a 78% efficacy against *P. falciparum* with *pfcrt* 76T in Guinea-Bissau [2]. We have also shown that the standard total dose prescribed and commonly taken is approximately 3 times the normal dose (75 mgkg^−1^) divided into smaller doses over 5 days [5]. It is therefore likely that *P. falciparum* with *pfcrt* 76T are generally treated effectively in Guinea-Bissau. Much of the selective advantage associated with *pfcrt* 76T during treatment with normal dose CQ [2], [14] is therefore lost unless absorption is poor or the prescribed treatment discontinued. This most probably accounts for the lack of cumulative increase of *pfcrt* 76T in Guinea-Bissau during the high transmission season.

The small, temporary, but significant increase of *pfcrt* 76T during the beginning of the high transmission season occurs at the same time as the number of treated children increases and approximately one month after the malaria incidence increases. *Pfcrt* 76T is likely to provide an advantage soon after treatment when CQ concentrations are moderately high [15]. An increased number of treated children will increase the chance of a new infection occurring in a child with a moderately high CQ concentration. This likelihood will also increases as the malaria incidence increases. Thus it is probable that the increased *pfcrt* 76T proportion is partly caused by selection of *pfcrt* 76T in new infections due both to an increased incidence of malaria and an increased number of treatments. However, the increased *pfcrt* 76T proportion is also likely to be partly due to recrudescence. *P. falciparum* usually recrudesce a few weeks after treatment [2] and the *pfcrt* 76T proportion should therefore increase a few weeks after an increase in malaria incidence, as it does. Despite this *pfcrt* 76T does not accumulate over the whole season supporting our hypothesis that only a fraction survive the high dose treatment used.

Monitoring prescription patterns in 2003–2004 showed that, out of 26134 consultations, 17924 (69%) of children below the age of 5 years were diagnosed with malaria while only 13% of these presumptively treated children had microscopically verified malaria [11]. Similar monitoring during 2007 and 2008 found that 13310/34884 (38%, monthly range 30–43%, authors unpublished data) of children (under 15 years) attending health centres were treated for malaria though only 415/13310 (3.2%, monthly range 0.4–8.9%, authors unpublished data) had microscopically verified malaria. In practice, CQ is used for the treatment of fever and the drug selective pressure is high all the year round. There is therefore no period of low CQ pressure during which *pfcrt* 76K has a distinct selective advantage as described in The Gambia and Sudan [9], [10]. This probably explains the surprisingly stable *pfcrt* 76T and *pfcrt* 76K proportions during the dry season.

Resistance is generally believed to develop from a sensitive parasite that first becomes tolerant and eventually resistant. Tolerance is a long process during which parasites acquire several genetic changes making them gradually more tolerant of CQ, whereas a resistant parasite is unaffected by drug exposure.[15]. *Pfcrt* 76T identifies a parasite that is very likely to be resistant when normal dose CQ is used, also in Guinea-Bissau [2], [14]. However, the common use of more efficacious high doses of CQ in Guinea-Bissau probably turns *pfcrt* 76T into a marker of tolerance. But, as discussed previously, *pfcrt* 76T is also associated with a loss of fitness. The probable explanation for the low and stable *pfcrt* 76T proportions is therefore the loss of fitness associated with *pfcrt* 76T that negates the advantage 76T provides when CQ concentrations are moderate. No parasite with the ability to survive the high doses of CQ routinely used has become established in Guinea-Bissau despite “tolerant” (*pfcrt* 76T) parasites existing since at least 1992 [4]. This suggests that development of further resistance is a difficult step in line with predictions [16] and the long time it took for CQ resistance to develop in the first place [17].

Mutations in *pfmdr1* have been associated with substantial fitness cost [18]. We therefore assessed parasite densities as a proxy marker of fitness. *Pfcrt* 76T and *pfmdr1* 86Y were associated with lower parasite densities suggesting a cost of fitness associated with at least one of these haplotypes as they are linked. This supports the fitness argument for why *pfcrt* 76T has reached fixation at a low prevalence in Guinea-Bissau. Including children above the age of 10 gave similar but not significant results. Differences in immunity might account for the age effect. However, the data should be interpreted with caution as the age effect is difficult to explain and because it is possible that *pfcrt* 76T identifies a more virulent parasite causing children to go to the health centres earlier.

As in the Gambia [10], there was no seasonal variation of *pfmdr1* 86N or 86Y proportions, there was linkage disequilibrium between *pfmdr1* 86Y and *pfcrt* 76T and no significant seasonal variation of the mean number of genotypes. The mean number of genotypes were lower in Guinea-Bissau suggesting less chance of sexual recombination in mosquitoes, that should slow the pace of resistance development [15].

As in The Gambia, we noted lower parasite densities during the second half of the high transmission season compared to the first half. This might be due to an enhanced malaria specific immunity. The differences in seasonal parasite density pattern between *pfcrt* 76T and *pfcrt* 76K parasites is difficult to interpret and is possibly due to small number of *P. falciparum* with *pfcrt* 76T (only 37) during the low transmission season.

In summary, the *pfcrt* 76T proportion does not gradually increase throughout the high transmission season and is low and stable between 2003 and 2007. We suggest that this is due to the use of a more efficacious dosage of CQ in Guinea-Bissau combined with a loss of fitness associated with *pfcrt* 76T. These factors largely remove the selective advantage that *P. falciparum* with *pfcrt* 76T have when normal dose CQ is used. The results therefore indicate that CQ can be an effective drug if dosed differently. As CQ is cheap and accessible further research into dosing strategies of CQ should be done.
